# Cancer Disparities Experienced by People with Disabilities

**DOI:** 10.3390/ijerph19159187

**Published:** 2022-07-27

**Authors:** Rosemary B. Hughes, Susan Robinson-Whelen, Carly Knudson

**Affiliations:** 1 Rural Institute for Inclusive Communities, University of Montana, Missoula, MT 59812, USA; carlyknudson11@gmail.com; 2 Spinal Cord Injury and Disability Research Center, TIRR Memorial Hermann, Houston, TX 77079, USA; susanrw@bcm.edu; 3 Department of Physical Medicine and Rehabilitation, Baylor College of Medicine, Houston, TX 77030, USA

**Keywords:** cancer, disability, disparities, risk factors, barriers to access

## Abstract

People with disabilities, who represent a rapidly growing and seriously disadvantaged segment of the U.S. population, face unremitting barriers to equal and accessible healthcare and a high prevalence of chronic health conditions. A slowly growing body of research suggests multiple cancer-related disparities between people with and without disabilities. This commentary identifies multiple aspects of the cancer experience and highlights ways cancer is impacted by disability. This includes vulnerabilities to risk factors, barriers to accessing healthcare, and disparities in screening, diagnosis, and treatment. The authors offer six essential pathways for reducing cancer disparities faced by people with disabilities. It is clear that reducing cancer health disparities experienced by people with disabilities will require the commitment and cooperation of a wide range of stakeholders.

## 1. Introduction

People with disabilities represent more than a billion people worldwide [1]. In the United States, an estimated 61 million, or one in four, noninstitutionalized adults live with a disability [2]. In addition, approximately two million people with disabilities live in institutional settings, such as prisons or skilled nursing homes, according to the ADA National Network [3]. Of note, people with disabilities under the age of 65 who live in such institutions comprise a percentage significantly greater than their counterparts without disabilities [4]. These numbers are anticipated to increase as a result of people worldwide living longer coupled with advances in medical science, pharmacology, and technology, allowing people with disabilities to enjoy improved survival rates and longevity. For the purposes of this manuscript, we subscribe to the World Health Organization’s broad definition of disability as involving an impairment that limits the ability of the person to execute certain activities and to participate in the world around them [5]. Although legal, health, and governmental entities conceptualize and define disability differently [5,6,7], there is a universal understanding that people with disabilities are disproportionately disadvantaged by marginalization and inequity and face attitudinal, environmental, systemic, communication, and other unremitting and significant barriers to equal and accessible healthcare [1,8]. In addition to experiencing multiple other disadvantages and inequities, people with disabilities may experience cancer health disparities.

Cancer is a leading cause of morbidity and mortality worldwide, accounting for an estimated 18.1 million cases in 2020 [9]. In the U.S. alone, over 1.9 million new cases of cancer are estimated to be diagnosed in 2022 [10]. Although there are published data on the numbers of beneficiaries receiving Social Security Disability Insurance due to cancer-related disability [11], there is no available systematic documentation on the incidence of cancer in people who had their disability before being diagnosed with cancer. However, as we demonstrate in this paper, a slowly growing body of research suggests that people with some types of preexisting disabilities have significantly higher rates of some types of cancer than people without those disabilities [12,13].

In this commentary, the authors do not conduct a comprehensive critical review but instead draw upon select empirical literature to highlight aspects of the cancer experience disproportionately impacted by disability. Such aspects include vulnerabilities to risk factors; barriers to accessing healthcare; and disparities in screening, diagnosis, and treatment. People with diverse disabilities, such as people who are deaf or hard of hearing [14], people with intellectual disabilities [15], and people with psychiatric disabilities [16], are known to experience cancer disparities.

## 2. Impact of Disability on Cancer

Vulnerabilities to Cancer Risk Factors. Advancing age, obesity, tobacco use, alcohol consumption, chronic inflammation, and unhealthy diet represent common or suspected risk factors for cancer [17]. According to the U.S. National Cancer Institute (NCI), “advancing age is the most important risk” for cancer overall [18]. The NCI reports on the later average age diagnosis of breast cancer, colorectal cancer, lung cancer, and prostate cancer [18]. The NCI also claims that chronic inflammation can cause DNA damage and lead to cancer [19], and that people with obesity may have an increased risk of several types of cancer, such as cancers of the breast in post-menopausal women and cancers of the colon [20]. People with disabilities report disproportionately higher rates of risk factors, including obesity [21], cigarette smoking [22], and substance misuse [23]. Moreover, chronic inflammation characterizes many disabling health conditions such as rheumatoid arthritis, multiple sclerosis, and systemic lupus erythematosus [24]. Additionally, people with disabilities often experience inadequate diet due to socioeconomic, environmental, and disability-related barriers, including greater difficulty shopping and preparing nutritional food [25].

Strong empirical evidence links low income, less education, and other socioeconomic inequalities with increased risks for cancer incidence, morbidity, mortality, and survival in the general population [26,27]. Although the relevant literature on socioeconomic status and cancer is somewhat complex and inconsistent, there is evidence for an association of low income and less education with a higher risk of cancer incidence. For example, an analysis of socioeconomic and racial/ethnic cancer-related disparities in the U.S. from 1950 to 2014 [28] indicated that people in more deprived areas or in lower education and income groups had higher cancer incidence and mortality rates than their more affluent counterparts, with the highest risk for lung, colorectal, cervical, stomach, and liver cancer. Additionally, according to an analysis of data on the impact of socioeconomic status on cancer incidence and stage of diagnosis from the National Longitudinal Mortality study [27], cancer patients diagnosed in 1973–2001 with less than a high school education had increased odds of lung cancer than those with a college education. Those with low family annual income also had higher lung cancer incidence than those with higher incomes. The presence of disability in a person’s life is linked with socioeconomic deprivation, including an increased likelihood of poverty or low income, lower levels of education, and unemployment [29,30,31]. This evidence suggests that households with people with disabilities experience socioeconomic insecurities associated with deprivations, including access to nutritional foods and access to health care, which can increase the risk for some types of cancer and hinder cancer diagnosis and survival.

Barriers to Cancer Screening and Care. Socioeconomic insecurities not only place one at greater risk for cancer, but they can also be an enormous barrier to cancer screening, diagnosis, and treatment. Research has documented that uninsured people with disabilities experience significantly more barriers to accessing healthcare than nondisabled persons without health insurance [32]. Many people with disabilities lack health insurance to cover the costs of cancer diagnosis, which can require multiple diagnostic tests. With disparate low employment rates, people with disabilities may lack employer-paid health insurance or the financial resources for private health insurance [33]. Moreover, their low-income levels often surpass the threshold for public insurance coverage. Even with health insurance, people with disabilities report unmet healthcare needs and barriers to accessing healthcare [34]. Advanced-stage cancer at diagnosis is associated with being uninsured in the general population [35,36]; thus, if uninsured or underinsured, people with disabilities may have to forego routine preventive care resulting in the diagnosis of a more advanced-stage cancer. According to the U.S. President’s 2022 Cancer Panel Report [37] (p. 2), “…gaps in cancer screening mean too many in the United St are unnecessarily enduring aggressive treatment or dying from cancers that could have been prevented or detected at earlier, more easily treated stages.”

There are many other barriers, in addition to socioeconomic barriers, that impact cancer prevention and care and contribute to cancer disparities. In a literature review on barriers to cancer screening in the context of disability [38], the authors identified *individual barriers*, such as anticipation anxiety regarding the procedure or inability to perform one’s own breast self-exam due to functional limitations [38]; *interpersonal barriers*, such as inadequate patient–provider communication; and *environmental barriers*, such as lack of reliable transportation to screening facilities and inaccessible medical equipment. These wide-ranging barriers can negatively impact cancer screening, diagnosis, and treatment, and thus ultimately impact survival.

Primary care providers often focus on the person’s disability to the exclusion of other preventive health matters [39]. One potential consequence of this is that women with disabilities are less likely than women without disabilities to receive a recommendation from a physician for mammography screening [40]. Providers may devalue or have negative perceptions about people with disabilities and lack disability-specific training and knowledge needed to accommodate the needs of their patients with disabilities [41,42,43]. For example, Iezzoni and colleagues [32] described the experience of a woman with tetraplegia whose primary care physician always examined her while seated in her wheelchair. However, another doctor discovered a breast lump when she was positioned on a table that was previously undetected in the seated exam. In a recent qualitative study [44], a woman with a physical disability reported that her doctor asked how she expected to be examined if she could not get up on the table. However, another participant shared that she could “see it in their eyes” as they looked at her in the wheelchair, looked at the height of the exam table, and then changed their minds about doing a Pap exam. Such interpersonal and environmental barriers can have devastating consequences for individuals living with disabilities who do not receive appropriate screening and thus do not receive an accurate, timely diagnosis.

Despite federal law requiring healthcare facilities in the U.S. to be accessible to people with disabilities [30], barriers persist such as the lack of access to entering and navigating healthcare facilities and the lack of a safe method for lifting, transferring, and positioning patients to an examination table [45]. According to the findings of a population-based study [34], more people with disabilities than those without disabilities experience difficulty obtaining an appointment, long wait times at the screening site, and lack of transportation to the clinic. Additionally, clinics may lack flexible mammography equipment with adjustable arm and height features to allow a person using a chair or a wheelchair to reach the machine; adjustable exam tables for colorectal and Pap screenings; or wheelchair platform scales [46].

Not only do people with disabilities experience barriers to screening services, but they also experience barriers to receiving an early cancer diagnosis which impacts chances of long-term survival [47]. In addition to experiencing transportation barriers and inaccessible facilities and diagnostic equipment, people with disabilities often encounter providers who fail to acknowledge their disability-related needs and provide disability-related accommodations; disregard cancer signs and symptoms as emotional responses to a chronic health condition; and erroneously attribute physical signs and symptoms of cancer to the underlying disability [48,49]. The barriers to accessing healthcare are exacerbated in a rural context [50]. Rural women with disabilities in the U.S. who require transportation and must manage greater distances to cancer screening providers or facilities, especially to those that provide accessible services, may be less likely than those who live in urban settings to receive breast and cervical cancer screening [51].

Disparities in Cancer Screening, Diagnosis, and Treatment. Given the multiple barriers to accessing healthcare, and particularly cancer screening, it is not surprising that research studies document disparities in various types of cancer screening or surveillance for people with disabilities including screening for breast, cervical, and colorectal cancer. A report published before the turn of the century on the use of cervical and breast cancer screening among U.S. women indicated that women with functional limitations were significantly less likely than women without functional limitations to have had a Pap test within the past three years [52]. This report also found that women 65 years old or older with three or more functional limitations were less likely to have ever had mammograms than women without disabilities of a similar age. Nearly a quarter of a century later, those disparities are still observed. An ever-increasing literature base leaves little doubt that women with disabilities continue to be less likely to receive breast and/or cervical cancer screening compared to women without disabilities [34,39,53,54,55,56,57,58,59,60,61,62,63]. Although the evidence is inconsistent regarding disparities in colorectal screening [12], some research suggests that people with disabilities may be less likely to have colorectal cancer screening than those without disabilities [56,64].

Disparities are not limited to cancer screening or surveillance rates. When compared to people without disabilities, people with disabilities have been found more likely to experience cancer [13]. While some research suggests people with disabilities receive a cancer diagnosis at advanced stages [65], other research indicates that, while they are diagnosed at similar or even earlier stages than people without disabilities, they experience disparate rates of cancer death when diagnosed at the same stage of cancer [66].

Recent research suggests that people with disabilities have significantly higher rates of some types of cancer. According to a population-based study on the association of cancer and disability [12], people with disabilities involving mobility and activity limitations had significantly higher rates of ovarian cancer, prostate cancer, colorectal cancer, and non-Hodgkin’s lymphoma than people without disabilities. Moreover, people with disabilities were significantly older on average than people without disabilities when diagnosed with those four types of cancer.

Growing evidence also suggests that people with disabilities experience inequities in cancer treatment and mortality [67]. Findings from a study on treatment for early-stage breast cancer among women under the age of 65 indicated that women with disabilities were less likely than other women to receive standard therapy (i.e., radiation therapy or axillary lymph node dissection) following breast-conserving surgery and experience higher breast-cancer-related and all-cause mortality rates [68]. More than a decade later, Ansmann and colleagues [69] reported that women with physical disabilities were more likely to undergo mastectomy than breast-conserving therapy, even after accounting for differences in age and socioeconomic status. Reasons for the disparities suggested by the authors included unequal access to treatment options; preferences such a desire for a briefer treatment process without radiation; individual difficulties such as limited transportation to clinics for radiation therapy; or complex comorbidities and other medical difficulties.

## 3. Pathways for Reducing Disparities

Throughout this brief commentary, we have considered cancer health disparities in the context of disability. Because the focus of cancer disparities experienced by people with a pre-existing disability has received minimal attention in the extant literature, there is little information on understanding the disparities, and much less on methods for reducing them. Clinical recommendations that have been made include conducting appropriate screening and follow-up, reducing the delay of diagnosis, and preventing practitioners from engaging in diagnostic overshadowing, that is, attributing potential signs of cancer to the person’s underlying disability [12]. We have cited evidence of differences between people with disabilities and people without disabilities in cancer screening, the timeliness of diagnosis, and the delivery of treatment. For a comprehensive list of barriers people with disabilities face when accessing cancer-related care and recommendations for improving access and care, we highly recommend a recent article by well-known disability and cancer researcher, Dr. Lisa Iezzoni [70]. Recognizing the inequities highlighted in this commentary, we offer six essential pathways for understanding and reducing cancer disparities facing people with disabilities.

1. Include culturally appropriate information about disability and cancer in the context of disability in medical and allied health education and training as well as continuing medical education programs. For example, medical schools could avail their students an opportunity to follow a newly diagnosed cancer patient with a pre-existing disability. Programs could also include a self-study exercise or a problem-orientated role-play and other interactive activities, whereby the student virtually treats a cancer patient with a disability under supervision. Such activities should be designed to reduce bias toward people with disabilities, increase knowledge regarding the shared and unique needs of people with disabilities, and develop a collaborative patient–provider relationship wherein the lived experience of disability informs their care;
2. Develop and test accessible and culturally tailored, community-based prevention and lifestyle change programs aiming to reduce potential cancer risks known to disproportionately impact people with disabilities such as physical inactivity, smoking, inadequate diet, and obesity;
3. Develop, implement, evaluate, and disseminate accessible, disability-relevant cancer health information for people with disabilities and providers on cancer in the context of disability. The online or print delivered information should encompass specific disability-related vulnerabilities to risks, barriers to healthcare, and disparities in screening, diagnosis, and treatment for people with disabilities. People with disabilities often have multiple types of disabilities. For example, a person with a mobility disability may also have a cognitive or sensory disability. Examples of ways to provide materials accessible to all include the use of plain, straightforward language; simple sentence structure; and a plain text version of materials with descriptions of images;
4. Advocate at local, state, and federal levels for legislation and the implementation of healthcare policies that address and reduce system barriers such as health care coverage limiting the ability of people with disabilities to access cancer-related resources and treatment; the built environment involving inaccessible service, facilities, and medical equipment; and the inequitable representation of people with disabilities in cancer research and clinical trials;
5. Using population-based datasets that include disability identifiers, conduct rigorous research designed to identify disability-related disparities in cancer risks, screening, prevention, and treatment. To address cancer disparities experienced by people with disabilities, studies conducted in the U.S. have analyzed population-based data from the Behavioral Risk Factor Surveillance System [53,55], the National Health Interview Survey [12,34,39,41,58,59,60,61], and the Medical Expenditure Panel Survey [51,57]. However, exploring disability-related cancer health disparities has historically been challenging given the limited information about disabilities and functional limitations contained in datasets typically used to examine cancer and cancer-related outcomes [67]. Given the high rates of service-connected disability in veterans [70] and recent data suggesting elevated rates of cancer among veterans [71], research is particularly needed on the intersection of disability and cancer in veterans using Veterans Administration databases;
6. Advocate for funding opportunities calling for high-quality research designed to: (a) advance the state of the science on cancer in the context of disability; (b) develop and test the effectiveness of interventions designed to reduce cancer disparities in people with disabilities; and (c) evaluate the efficacy of interventions beyond controlled conditions to routine settings and document feedback from providers and patients to determine their real-life effectiveness and sustainability.

## 4. Conclusions

People with disabilities are a rapidly growing, marginalized population that experience cancer health disparities stemming from disability-related deficits in the training of medical professionals, the pervasive inaccessibility of medical facilities and equipment, and limitations in health insurance and healthcare policies. This brief commentary represents a snapshot of some disability-related cancer disparities; however, it is limited in scope as it emphasizes research addressing cancer in people with physical disabilities. This is not meant in any way to minimize cancer disparities experienced by people with intellectual, developmental, sensory, psychiatric, or other disabilities. As is evident by the six essential pathways we have outlined, addressing and reducing disability-related cancer health disparities will be challenging. It will require the commitment and cooperation of a wide range of stakeholders. It is imperative that governmental agencies, healthcare providers, people with disabilities and their advocates, research professionals, and other stakeholders establish partnerships with an overriding goal to increase the understanding of cancer in the context of disability and reduce cancer health inequities experienced by people with disabilities.
